# Manual lymphatic drainage before and after total knee arthroplasty, a randomized controlled trial

**DOI:** 10.1016/j.jcot.2024.102401

**Published:** 2024-04-14

**Authors:** Moritz Wagner, Andreas Wittlinger, Alexander Auffarth, Franz Endstrasser, Sabrina Neururer, Alexander Brunner

**Affiliations:** a Department of Orthopaedics and Traumatology, Bezirkskrankenhaus, Bahnhofstrasse 14, 6380 St. Johann, Tyrol, Austria; b Department of Orthopedics and Traumatology, Paracelsus Medical University Salzburg, Muellner Hauptstr. 48, 5020, Salzburg, Austria; c Lymphklinik Wittlinger Therapiezentrum GmbH, Austria; d Department of Clinical Epidemiology, Tyrolian Federal Institute for Integrated Care, Tirol, Austria; e Medical University Innsbruck, Tirol, Austria

**Keywords:** TKA, Manual lymphatic drainage, MLD, Swelling, Outcome, Surgery, Knee

## Abstract

**Purpose:**

Manual lymphatic drainage (MLD) is a routine therapeutic technique used to decrease peripheral oedema by activating lymphatic drainage. Evidence for its efficacy remains sparse. Therefore**,** the purpose of this study was to evaluate the effect of MLD before and after total knee arthroplasty (TKA).

**Methods:**

This was a single-centre randomized, controlled and observer-blinded trial. 112 patients were randomly assigned to one of three groups: group 1 underwent MLD for 30 min daily on five consecutive days both before and after TKA; group 2 underwent MLD for 30 min daily on five consecutive days only after TKA; and the control group did not undergo MLD. The Western Ontario and McMaster Universities Osteoarthritis Index (WOMAC) score, range of knee motion, swelling and pain were assessed before TKA, after two days, five days and six weeks.

**Results:**

The overall age of the patients was 69.4 years (SD = 9.8, range = 41–87). The groups were well matched in terms of sex, age, height, weight, and body mass index. There was no statistically significant difference with regard to any of the outcome measures between the groups.

**Conclusions:**

The present results indicate that MLD had no significant benefits when applied either before or early after TKA. Therefore, we do not recommend the routinely use of MLD in the early period before or after TKA. Further studies should evaluate the effect of MLD after arthroscopic surgery.

**Level of evidence:**

Therapeutic Level II, Lower quality RCT with follow up <80 %

## 1 Introduction

Manual lymphatic drainage (MLD) is a routine therapeutic technique used to decrease peripheral oedema by activating lymphatic drainage.^1^, ^2^, ^3^ During the last two decades, it has been commonly accepted as a standard postoperative treatment for orthopaedic surgical procedures.^1^ Especially in patients who have underdone total knee arthroplasty (TKA), MLD is considered to be useful for achieving early reduction of postoperative swelling, improving early range of motion (ROM), and minimizing the risk for wound healing complications.^4^, ^5^, ^6^ Therefore, MLD is often performed along with the application of elastic bandages and cryotherapy.^5^ However, according to recent medical literature, neither bandages nor cryotherapy have shown a significant effect on the postoperative outcome.^4^^,^^5^ With regard to MLD, the general consensus is that it is a safe procedure, but data about its actual impact on postoperative swelling, ROM and pain remain sparse. Further, existing studies on the effects of MLD after TKA are under-powered^3^^,^^7^ or lack a non-interventional control group.^2^ However, despite the lack of evidence for the efficacy of this method, a high number of MLD procedures are performed each year and are also associated with significant healthcare costs.

The purpose of this study was to evaluate the effect of MLD before and after TKA on patient-reported outcomes, swelling, ROM and pain. Our primary research hypothesis was that MLD before and after TKA leads to improved patient-reported outcome (according to the Western Ontario and McMaster Universities Osteoarthritis Index [WOMAC]), and our secondary hypothesis was that MLD reduces postoperative pain and swelling and improves ROM.

## 2 Patients and methods

### 2.1 Patient population and surgery

This was a registered single-centre randomized, controlled and observer-blinded trial (registered as trial number NCT05119764 with the U.S. National Library of Medicine). Institutional review board approval was obtained prior to conducting this study (approval number 1466/2020, Ethikkommission Innsbruck). The study was conducted at the Department for Orthopaedic and Trauma Surgery at the Bezirkskrankenhaus St. Johann in Tirol (Austria) between March 2021 and July 2022. Patients who had symptomatic osteoarthritis of the knee and were scheduled for primary TKA were included. The exclusion criteria were previous study participation with TKA on the other side, severe obesity with BMI >35, lymphatic oedema, vascular diseases, and intraoperative complications, including those which necessitated semi-constrained and rotating-hinge implant placement.

The surgical procedures were carried out by five experienced arthroplasty surgeons. According to the preferences of the surgeons, either the Attune Knee System or the LCS Complete Knee System (Johnson & Johnson, New Jersey, USA) with cemented femoral and tibial components and mobile-bearing liners was used. The surgical approaches varied according to the patients’ pathology and included the medial parapatellar and lateral parapatellar approaches. The tibia was dissected first, and the knees were ligament-balanced in all cases. The use of a tourniquet and the application of tranexamic acid varied between the cases, however there was no systematic bias. As part of the standard procedure, a continuous femoral nerve catheter was placed in all patients during the first two postoperative days. The rehabilitation protocol was initiated on the first postoperative day and included the use of a continuous passive motion machine and conventional physiotherapy with assisted mobilization. The minimum length of hospital stay was 5 days. The discharge criteria were a sealed and dry surgical wound, minimum active knee flexion of 90°, and the ability to independently walk up a flight of stairs.^6^

### 2.2 Interventions

Patients were randomly assigned to one of three groups by simple randomization without stratification: (1) the pre- and postoperative MLD group, which underwent 30 min of MLD daily for five consecutive days both before and after surgery; (2) the postoperative MLD group, which received MLD daily for five consecutive days after surgery and (3) the control group, which did not receive MLD. MLD was performed by specially trained physiotherapists from an external facility. The MLD technique was performed according to the recommendations of Dr. Emil Vodder from Denmark, who invented this technique in the early 1930s. Along with Günther Wittlinger, he described five basic movements: (1) scooping, (2) pumping, (3) stationary circles, (4) thumb circling and (5) rotary movements.^8^ Only the first four movements were used for the lower extremity. The treatment is initiated from the so-called ‘short neck’ and is performed from the proximal to distal direction. Preoperative MLD was performed at the private homes of the patients, and postoperative MLD was performed in the surgical ward of the hospital. During their inhouse stay, patients from different groups were accommodated in separate rooms to minimize the risk of nonadherence.

### 2.3 Outcome measures

**Fig. 1:**
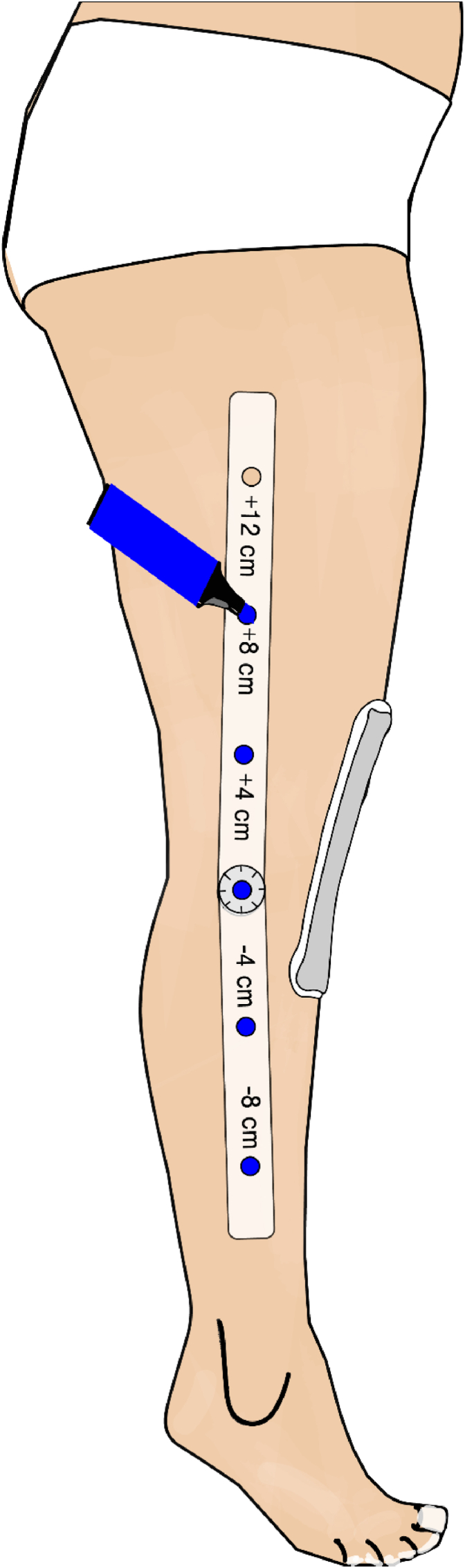
Schematic illustration of exact marking points distal and proximal to the joint line with a goniometer that has holes through which ink is applied to the skin.

**Fig. 2:**
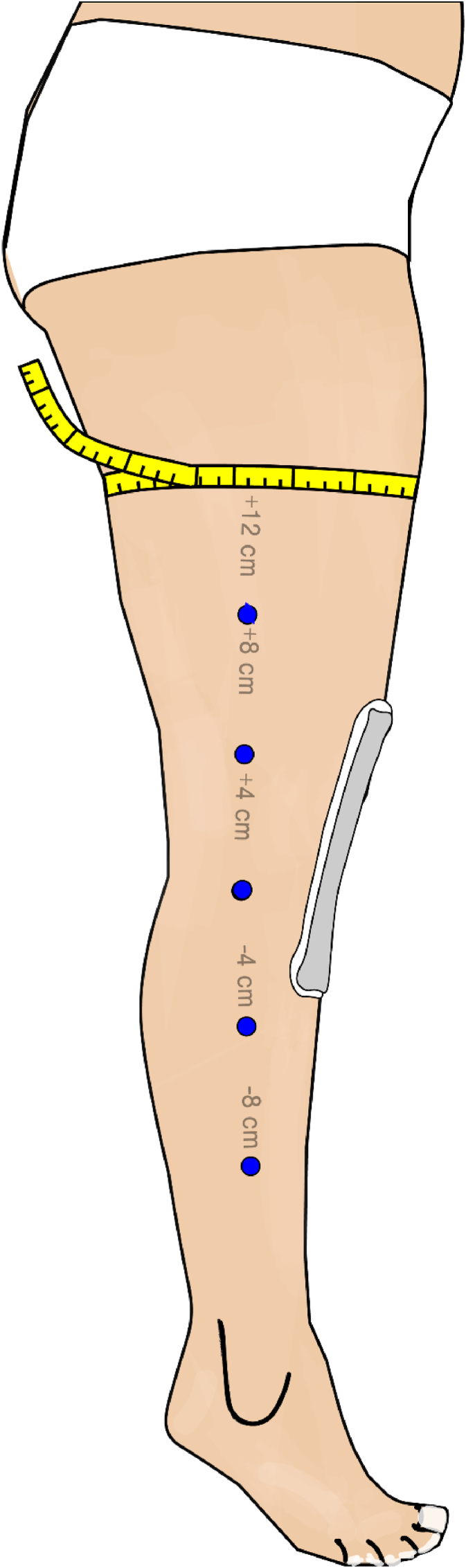
Schematic illustration of the circumferential measurements at the marked points with a tape measure that is applied without pressure.

The primary outcome measure was the WOMAC score, calculated based on a questionnaire filled up on paper by the patients. The WOMAC score was scaled from 0 (best) to 100 (worst), which was assessed 2 weeks before surgery and 5 days after surgery. The secondary outcome included the level of pain, swelling, and active and passive ROM. Pain was scored from 0 to 10 on a visual analogous scale, and ROM was assessed with a goniometer. The same goniometer had pre-drilled holes at 4-cm distances. The goniometer was placed at the joint line and those pre-drilled holes used to mark the skin with permanent ink (Fig. 1). In a second step, the goniometer was removed, and a flexible tape measure was used to assess the circumference of the leg on those six pre-marked points (Fig. 2). Swelling was then calculated with a mathematical formula and recorded as volume in litres based on the six standardized circumferential measurements around the calf, knee, and thigh.^9^^,^^10^ All secondary outcome parameters were assessed 2 weeks before surgery, 1 day before surgery, 2 days after surgery, 5 days after surgery, and 6 weeks after surgery by independent in-house physiotherapists who had not been involved with the MLD procedure and were blinded to prior interventions. Initially, a pilot study was conducted on six patients who did not undergo any interventions to evaluate the feasibility of the planned trial and to optimise the set-up and workflow process. Multiple measurements were carried out on the same patients to increase intra- and inter-rater reliability.

### 2.4 Statistical analysis

Sample size calculation was based on a power analysis that used a validated minimum clinically important difference of 10 points for the primary outcome measure (WOMAC score).^11^ Power analysis was performed using one-way analysis of variance (ANOVA) with repeated measures between factors, with an effect size (f) of 0.25 and an error probability (p) of 0.05, for three groups with five measurements. This resulted in a minimum total sample size of 96 patients. To ensure that the study power was not affected by patients dropping out later on in the study, we defined the minimum sample size of each group as 36 patients, and this was equivalent to 112 % of the required sample size.

Descriptive statistics of numerical variables were presented as means with standard deviations and ranges. Categorical variables were presented as absolute and relative frequencies in percentages. Comparison between groups was performed with one-way ANOVA for numerical variables and the chi-square test for categorical variables. The level of statistical significance was set at p < 0.05 for all tests. Statistical evaluation of the data was performed with the help of a biomedical statistician using the SPSS software (version 27; SPSS Inc., Chicago, USA).

## 3 Results

**Fig. 3:**
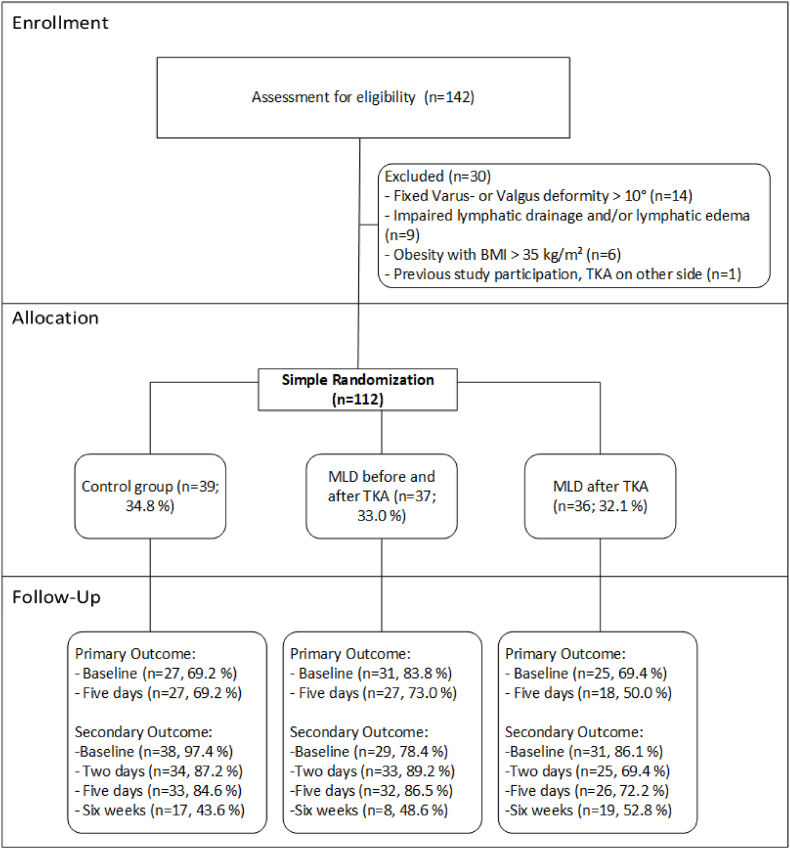
Diagram depicting patient enrolment, allocation and follow-up of this randomized controlled trial assessing the effect of manual lymphatic drainage before and after total knee arthroplasty, following CONSORT recommendation. Abbreviations: TKA: total knee arthroplasty; MLD: manual lymphatic drainage.

A total of 142 patients fulfilled the inclusion criteria, but 30 patients were excluded. The remaining 112 patients were divided into the control group (n = 39), the pre-/postoperative MLD group (n = 37), and the postoperative MLD group (n = 36). The protocol for patient enrolment, allocation and follow-up is summarized in the CONSORT flow chart in Fig. 3. There was a strict adherence to the treatment protocol, regarding patient allocation. No cross-over between groups occurred. One third of the measurements could not be carried out, therefore an intention to treat analysis was carried out to accommodate for loss of data (Fig. 3).

The overall age of the patients was 69.4 years (SD = 9.8, range = 41–87). Of the total patients, 66 were women (58.9 %) and 46 were men (41,1 %). The groups were well matched in terms of sex, age, height, weight, and BMI (Table 1).

**Table 1:** Distribution of age, height, weight and BMI between groups.

	Control group (n = 39)	MLD before and after TKA (n = 37)	MLD after TKA (n = 36)	Total (n = 112)	p value
Age (years)	70.9 (SD = 9.3)	67.7 (SD = 8.5)	69.8 (SD = 11.4)	69.4 (SD = 9.8)	0.393
Height (cm)	167.7 (SD = 8.7)	166.4 (SD = 9.3)	169.7 (SD = 10.0)	167.8 (SD = 9.3)	0.435
Weight (kg)	80.1 (SD = 15.1)	82.8 (SD = 15.0)	81.6 (SD = 20.7)	81.5 (SD = 16.7)	0.838
BMI	28.5 (SD = 12.3)	29.9 (SD = 12.2)	28.3 (SD = 13.1)	28.9 (SD = 12.4)	0.637

Abbreviations: TKA: total knee arthroplasty; MLD: manual lymphatic drainage; BMI: body mass index.

**Fig. 4:**
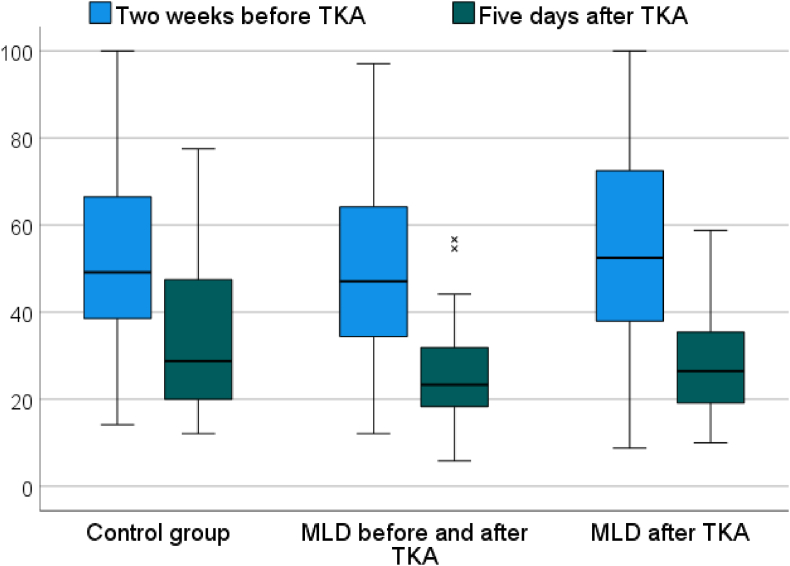
Box-plot depicting WOMAC scores (0 = best, 100 = worst) for each group before and five days after total knee arthroplasty. Abbreviations: TKA: total knee arthroplasty; MLD: manual lymphatic drainage.

**Fig. 5:**
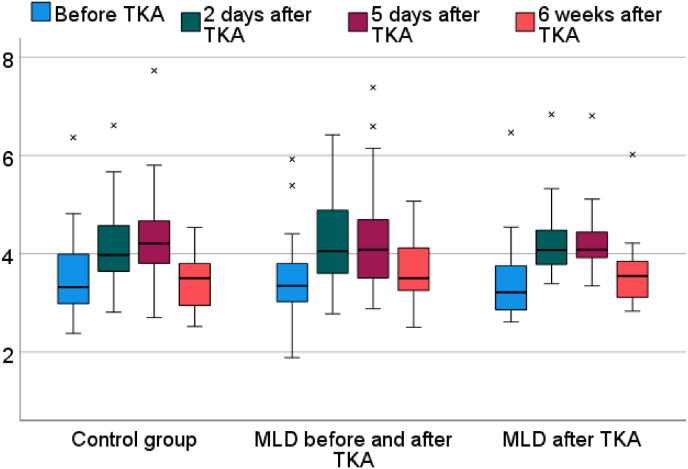
Box-plot of leg volume (in litres) for each group at multiple time points before and after total knee arthroplasty Abbreviations: TKA: total knee arthroplasty; MLD: manual lymphatic drainage.

There was no statistically significant difference with regard to any of the outcome measures between the groups. The WOMAC scores improved from 51.3 (SD = 21.6) before surgery to 29.9 (SD = 14.9) to 5 days after surgery, but there were no significant differences between the groups (Fig. 4). The results of volume measurement showed most severe swelling at day 2 and day 5 after surgery (Fig. 5). Active and passive knee flexion improved gradually over time. The level of pain improved from 5.3 (SD = 2.4) before surgery to 3.1 (SD = 2.3) on postoperative day 5 and to 2.5 (SD = 2.2) at postoperative week 6. There were no statistically significant differences between groups regarding any outcome measurement. The data for the secondary outcome parameters are summed up in Table 2.

**Table 2:** Mean leg volume, active knee flexion and pain score at various timepoints.

		Control group (n = 39)	MLD before and after TKA (n = 37)	MLD after TKA (n = 36)	Total (n = 112)	p value
Before surgery	**Volume**	3.4 (SD = 0.8)	3.4 (SD = 0.8)	3.4 (SD = 0.7)	3.4 (SD = 0.8)	0.925
	**Flexion**	119.6 (SD = 14.7)	117.6 (SD = 14.9)	119.1 (SD = 14.9)	118.9 (SD = 14.7)	0.862
	**Pain**	5.0 (SD = 2.5)	5.4 (SD = 2.2)	5.4 (SD = 2.3)	5.3 (SD = 2.3)	0.714
2 days after surgery	**Volume**	4.0 (SD = 0.8)	4.1 (SD = 0.9)	4.2 (SD = 0.7)	4.1 (SD = 0.8)	0.802
	**Flexion**	60.4 (SD = 23)	54.4 (SD = 20.1)	61.9 (SD = 22.8)	58.7 (SD = 21.9)	0.424
	**Pain**	3.2 (SD = 2)	3.3 (SD = 2.3)	4.2 (SD = 2.8)	3.5 (SD = 2.3)	0.303
5 days after surgery	**Volume**	4.2 (SD = 0.9)	4.2 (SD = 1)	4.2 (SD = 0.6)	4.2 (SD = 0.9)	0.980
	**Flexion**	71.2 (SD = 19.5)	70.2 (SD = 16.6)	75 (SD = 21.4)	71.9 (SD = 19)	0.633
	**Pain**	3.2 (SD = 2.7)	2.6 (SD = 1.5)	3.7 (SD = 2.3)	3.1 (SD = 2.2)	0.186
6 weeks after surgery	**Volume**	3.4 (SD = 0.6)	3.5 (SD = 0.7)	3.6 (SD = 0.7)	3.5 (SD = 0.6)	0.810
	**Flexion**	103.5 (SD = 12.8)	104.1 (SD = 16.2)	101 (SD = 27.6)	102.8 (SD = 19.9)	0.885
	**Pain**	2.8 (SD = 2.3)	2.2 (SD = 1.9)	2.5 (SD = 2.3)	2.5 (SD = 2.1)	0.723

Volume is expressed in litres, flexion in degrees, and pain as the score from a scale of 0 (no pain) to 10 (extreme pain).

Abbreviations: TKA: total knee arthroplasty; MLD: manual lymphatic drainage.

## 4 Discussion

The most important findings were that MLD before or early after TKA had no effects on swelling, range of motion or pain. Unfortunately, this study was slightly under-powered, due to a high rate of rate of patients, that were lost to follow-up at six weeks during the COVID-19 pandemic. However, this was still the largest randomized controlled trial, to assess the efficacy of MLD for patients who have undergone orthopaedic surgery.

The method of calculating leg volume by multiple circumferential measurements is commonly used to assess swelling in clinical practice in the field of plastic surgery (Fig. 1).^9^ This method is reliable and reproducible, and has been used in all previous studies on this subject.^3^^,^^7^^,^^12^ Therefore, this method was used in this study, too, and demonstrated an increase in leg volume around the knee from around 3.45 L before surgery to 4.17 L at day 2 after surgery, and it further increased to 4.24 L at day 5 after surgery. Leg volume was nearly normal at 6 weeks after surgery (3.57 L versus 3.45 L [before surgery]).

MLD is commonly deployed for the management of idiopathic lymphedema and lymphedema after cancer treatment, and its effects have been investigated on a large scale for oncologic patients. Meta-analyses and a Cochrane review on this subject demonstrated minor volume reduction with MLD in a subgroup of patients with mild lymphedema in the early stages following breast-cancer treatment, but MLD had no effect on patients with moderate-to-severe lymphedema after breast cancer treatment.^13^, ^14^, ^15^ The previous studies mainly assessed upper extremity lymphedema. Nevertheless, there might be a relationship in the efficacy of MLD according to the amount of swelling. Those studies for idiopathic lymphedema with severe disease progression showed no effect of MLD, likewise our study with MLD after TKA, which is an orthopedic procedure resulting in severe swelling, showed no effect.

Literature on the effects of MLD on the outcomes of orthopaedic procedures remains sparse. Four randomized, controlled and observer-blinded clinical trials, all of which measured the effect of MLD after TKA with similar methodology, have been carried out previously.^3^^,^^7^^,^^12^^,^^16^ In one of these trials, Fujiura et al. reported that there were no differences in pain and ROM in a cohort of 41 patients.^12^ Further, in Ebert et al.’s study, which included 43 patients, no differences were found in swelling and pain, but a 10° improvement was detected in active knee flexion. However, the measurements were carried out directly after MLD. This potentially limits the significance of the results, as data collected over a longer time period after MLD would be more meaningful than data taken over just a few minutes after MLD.^7^ In the third such trial, Pichonnaz et al. studied 60 patients and found no differences in swelling or ROM, but they observed a significant reduction in pain a few minutes after the manual lymphatic drainage.^3^ Finally, in the fourth study, Tornatore et al. compared MLD combined with kinesiotaping to kinesiotaping alone in 99 patients, and the results revealed improved leg circumference and less pain with MLD but no effects on ROM.^16^ In summary, the results from previous studies were heterogenous and found little to no effect of MLD. However, in all these studies, the effects of MLD were assessed on relatively small sample sizes, and outcome measures were obtained only minutes after the intervention. A recent literature review has evaluated the effects of MLD after orthopedic procedures in general, concluded that MLD should be reserved only for persistent lymphedema after elective surgery and not routinely deployed.^17^ In the present study, sample size was reached according to power-analysis, furthermore the effects of MLD before TKA were assessed. Outcome measures during the in-hospital stay were obtained many hours after the interventions.

The preventive effects of MLD before surgery have never been scientifically investigated in patients who have undergone TKA or any other orthopaedic procedure. However, three randomized controlled trials on a population of oncology patients that measured the preventive effects of MLD on the complications of breast cancer treatment have been conducted in the past. Two of these trials found that MLD did not have any preventive effects^18^^,^^19^, but one of them found a reduction in arm volume with MLD.^15^ This study adds to the literature by assessing the influence of MLD on patient-reported outcome, ROM and swelling after TKA in a large cohort. This is the first study to assess the effects of MLD before surgery, and we believe that the results may be relevant for major orthopaedic surgery of the lower extremities.

The major limitation of our trial is the high number of patients who were lost at the six-week follow up and some patients with the lack of baseline data, even though the necessary sample size was reached according to a-priori power-analysis. This occurred because of restrictions on ongoing clinical trials during the COVID-19 pandemic and precautions taken to limit the spread of the virus. Furthermore, there was no funding available for this study, therefore no study nurse could be employed to check for completeness of the gather data. Another limitation is that the patients in the control group were not blinded to the treatment, as they received no placebo therapy, for example a simple massage without lymphatic decongestion. Last, there was a limited follow-up duration of six weeks. It is evident from most rehabilitative measures, that effects endure usually only in the early postoperative period until three months after surgery, and evidence from other trials on the effects of MLD showed no long-term effects, therefore the long-term outcome has not been included in the evaluation.

## 5 Conclusion

The present results indicate that MLD had no clinically relevant benefits when applied either before or early after TKA. Therefore, we do not recommend the routinely use of MLD in the early period before or after TKA. Further, in the future, it is important to investigate whether patients with minor swelling after orthopaedic procedures, including arthroscopic surgery, may benefit from MLD, and if there exist beneficial effects if MLD is applied in the later period after TKA.

## Ethics approval

The Ethical Committee of the Medical University Innsbruck has approved this study, number 1466/2020.

## Trial registration

This trial was registered with ClinicalTrials.gov. ID: NCT05119764, Unique Protocol ID: 1466/2020.

## Source(s) of support

This research did not receive any specific grant from funding agencies in the public, commercial, or not-for-profit sectors. The authors received no financial support for the research, authorship, and/or publication of this article.

## Use of artificial intelligence

There was no use of artificial intelligence during any phase of this study. This manuscript was written without any support from artificial intelligence such as large language models.

## Credit author contribution statement

Moritz Wagner: Conceptualization (Lead), Methodology (Lead), Formal analysis and investigation (Lead), Writing - original draft preparation (Lead), Writing - review & editing (Lead), Correspondence (Lead) - Overall lead in conceptualizing and designing the study, leading the analysis, drafting and revising the manuscript, and serving as the corresponding author for all communications related to the manuscript.

Andreas Wittlinger: Methodology and Intervention (Supporting) - Contributing to the development and implementation of the methodology, responsible for manual lymphatic drainage protocols and organization of therapy intervention.

Alexander Auffarth: Methodology (Supporting) - Assisting in developing and planning the experimental setup and data collection protocols.

Franz Endstrasser: Data Curation (Supporting) - Assisting in managing and verifying the underlying data relevant to the study.

Sabrina Neururer: Resources (Supporting), Visualization (Supporting) - Assisting in the visualization of data and findings.

Alexander Brunner: Conceptualization (Supporting), Writing - review & editing (Supporting) - Contributing to the generation of the study's main ideas and objectives, and providing critical review and editing of the manuscript.

## Declaration of competing interest

The authors declare that they have no known competing financial interests or personal relationships that could have appeared to influence the work reported in this paper.
